# Obesity paradox in joint replacement for osteoarthritis — truth or paradox?

**DOI:** 10.1007/s11357-021-00442-x

**Published:** 2021-08-27

**Authors:** Setor K. Kunutsor, Michael R. Whitehouse, Ashley W. Blom

**Affiliations:** 1 National Institute for Health Research Bristol Biomedical Research Centre, University Hospitals Bristol and Weston NHS Foundation Trust and the University of Bristol, Bristol, UK; 2 Translational Health Sciences, Bristol Medical School, Musculoskeletal Research Unit, University of Bristol, Learning & Research Building (Level 1), Southmead Hospital, Bristol, BS10 5NB UK

**Keywords:** Obesity paradox, Cardiovascular disease, Osteoarthritis, Hip replacement, Knee replacement

## Abstract

Obesity is associated with an increased risk of cardiovascular disease (CVD) and other adverse health outcomes. In patients with pre-existing heart failure or coronary heart disease, obese individuals have a more favourable prognosis compared to individuals who are of normal weight. This paradoxical relationship between obesity and CVD has been termed the ‘obesity paradox’. This phenomenon has also been observed in patients with other cardiovascular conditions and diseases of the respiratory and renal systems. Taking into consideration the well-established relationship between osteoarthritis (OA) and CVD, emerging evidence shows that overweight and obese individuals undergoing total hip or knee replacement for OA have lower mortality risk compared with normal weight individuals, suggesting an obesity paradox. Factors proposed to explain the obesity paradox include the role of cardiorespiratory fitness (“fat but fit”), the increased amount of lean mass in obese people, additional adipose tissue serving as a metabolic reserve, biases such as reverse causation and confounding by smoking, and the co-existence of older age and specific comorbidities such as CVD. A wealth of evidence suggests that higher levels of fitness are accompanied by prolonged life expectancy across all levels of adiposity and that the increased mortality risk attributed to obesity can be attenuated with increased fitness. For patients about to have joint replacement, improving fitness levels through physical activities or exercises that are attractive and feasible, should be a priority if intentional weight loss is unlikely to be achieved.

Overweight and obesity are terms that refer to an excess of body fat and they usually relate to increased weight-for-height (World Health Organization Fact Sheets. https://www.who.int/news-room/fact-sheets/detail/obesity-and-overweight. Accessed on 29 March 2021). The most common method of measuring obesity, and that recommended by the World Health Organisation, is the body mass index (BMI). In adults, overweight is defined as BMI of 25 to 29.9 kg/m^2^ with a BMI of 30 kg/m^2^ or higher being classified as obese (World Health Organization Fact Sheets. https://www.who.int/news-room/fact-sheets/detail/obesity-and-overweight. Accessed on 29 March 2021). Some guideline bodies such as the UK National Institute for Health and Clinical Excellence (NICE) recommend the use of BMI in addition to waist circumference as the method of defining overweight and obesity and determining health risks (Statistics on Obesity, Physical Activity and Diet, England, 2020. https://digital.nhs.uk/data-and-information/publications/statistical/statistics-on-obesity-physical-activity-and-diet/england-2020/part-3-adult-obesity-copy. Assessed on 26 March 2021). In 2016, more than 650 million adults worldwide (13%) were reported to be obese (World Health Organization Fact Sheets. https://www.who.int/news-room/fact-sheets/detail/obesity-and-overweight. Accessed on 29 March 2021); obesity has reached epidemic levels in most Western countries. It is reported that 75% of the US population is either overweight or obese [30]. In the UK, it was reported that 63% of adults were either overweight or obese in the year 2018 (Statistics on Obesity, Physical Activity and Diet, England, 2020. https://digital.nhs.uk/data-and-information/publications/statistical/statistics-on-obesity-physical-activity-and-diet/england-2020/part-3-adult-obesity-copy. Assessed on 26 March 2021).

Obesity has adverse effects on health outcomes, healthcare systems and economies [30]. Obesity is a major risk factor for vascular disease, non-vascular disease and all-cause mortality. Obesity is strongly associated with increased risk of cardiometabolic conditions such as hypertension, metabolic syndrome, diabetes mellitus, coronary heart disease (CHD), heart failure (HF), and atrial fibrillation (AF) [39]. Obesity is also associated with increased risk of cancers [4]. In the midst of the current pandemic attributed to coronavirus disease 19 (COVID-19), obesity has been linked with an increased risk of critical illness and death due to COVID-19 [43]. Obese individuals are at increased risk of musculoskeletal conditions such as osteoarthritis (OA) in both weight- and non-weight–bearing joints. Obesity is known to double the lifetime risk of knee OA [35] and increases the risk sevenfold when compared to people with a BMI < 25 [34]; obesity also increases the risk of hand OA twofold compared with people with a BMI of 25–25 kg/m^2^ [14]. The relationship between obesity and OA has been described as strong and causal [50].

Paradoxically, despite the adverse effects of obesity on health outcomes, there are findings of an unusual relationship between BMI and outcomes in HF patients. In patients with already established HF, a wealth of evidence suggests that overweight and obese individuals have substantially improved survival rates compared with those with normal BMI [8],Lavie et al. 2016a). This phenomenon has led to the concept termed “reverse epidemiology” or “obesity paradox” (Lavie et al. 2016b). The obesity paradox has also been observed in patients with other cardiovascular conditions such as acute coronary syndromes (ACSs), CHD and AF, end-stage renal disease, respiratory conditions such as chronic obstructive pulmonary disease and pulmonary embolism as well as infections of varied sources (sepsis, pulmonary, urinary, and skin) [13, 29, 32, 38, 44].

Total joint replacement, one of the most common elective surgical procedures performed worldwide, is a highly successful and cost-effective intervention for alleviating pain and disability associated with advanced joint disease such as OA [10, 12]. The body of evidence suggests that obese people are more likely to undergo total hip or knee replacement (THR and TKR) [16], which is performed to treat end-stage OA in the overwhelming majority of cases. It is well established that there is a strong relationship between OA and CVD. Patients with OA are about three times more likely to develop CVD or HF [15] and approximately half of all adults with CVD also have a form of arthritis. There is abundant data showing that obese patients undergoing total hip or knee replacement have an increased risk of complications such as infection, venous thromboembolism, dislocation, and reoperations [2, 21, 23]. However, there is emerging evidence which shows that overweight and obese individuals undergoing TJR for OA have lower mortality risk compared with normal weight individuals, suggesting an obesity paradox (Table 1). Data based on the National Joint Registry for England, Wales, Northern Ireland and the Isle of Man showed that, compared with patients with normal BMI, the risk of 90-day mortality following THR for OA was lower in BMI categories — overweight, obese class I, and obese class II; whereas underweight patients had a substantially higher risk of mortality [17, 37]. Recent findings from the same registry which was based in TKR patients showed that overweight and obese class I were associated with a lower 90-day mortality rate than normal BMI [11]. In analyses based on two arthroplasty registries (the St Vincent's Melbourne Arthroplasty (SMART) Registry from Australia and the Kaiser Permanente Total Joint Replacement Registry (KPTJRR) from the USA), with more than 4 years median follow-up, being underweight was associated with higher all-cause mortality, whereas overweight, obese class I and obese class II were at a lower risk of mortality after total hip and knee replacement for OA when compared with normal-weight patients [9]. In a single-centre study of 756 primary THR replacements, high BMI was protective of mortality [18]. Data from Danish nationwide registries comprising patients who underwent elective primary hip or knee replacement surgery demonstrated high mortality among underweight patients, with the lowest risk of mortality among overweight patients [49].

**Table 1:** Observational studies assessing the association between body mass index and mortality following hip or knee replacement

Author, year of publication	Location	Population source	Baseline average age	Follow-up duration (yrs)	Joint/number of joints/patients	Major indication for surgery	Outcome	Risk estimates (95% CIs) for mortality	Covariates adjusted for
Hunt et al. 2013 [17]	UK	NJR	NR	8.0	THR/409,096	OA	90-day mortality	*Normal weight (ref):*Underweight: 1.71 (0.99–2.95); Overweight: 0.76 (0.62–0.92);BMI > 30 kg/m2: 0.88 (0.71–1.10)	Age, sex, comorbidity, year of operation, ASA, approach, prophylaxis (mechanical and chemical), anaesthetic and hip type/bearing
Jamsen et al. 2013 [18]	Finland	Joint replacement database	80	5.0	TKR/1,242THR/756	OA	All-cause mortality	**TKR**Per unit increase: 0.99 (0.96–1.03)**THR**Per unit increase: 0.91 (0.86–0.96)	Age, sex, operated joint, laterality, and anaesthesiologic risk score
Thornqvist et al. 2014 [49]	Denmark	Danish Nationwide Registries	5–75	1.0	TKR and THR /34,744	NR	1-year mortality	*Overweight (ref):*Underweight: 5.20 (3.50–7.80)Normal weight: 1.60 (1.30–2.00)Obese 1: 1.10 (0.87–1.40)Obese 2: 1.40 (1.01–2.00)	Age, gender, and hip vs. knee replacement surgery, heart failure, previous MI, chronic ischemic heart disease, atrial fibrillation, peripheral artery disease, cerebrovascular disease, chronic obstructivepulmonary disease, renal disease, diabetes, and prosthesis type
Mouchti et al. 2018 [37]	UK	NJR	NR	11.8	THR/413,741	OA	90-day mortality	*Normal weight (ref):*Underweight: 2.09 (1.51–2.89); Overweight: 0.70 (0.61–0.81);Class I obese: 0.69 (0.59–0.81);Class II obese: 0.79 (0.63–0.98);Class III obese: 0.73 (0.51–1.05)	Age, sex, ASA grade, and year of operation with stratification for indication for operation
Dowsey et al. 2018 [9]	Australia	SMART Registry	71	5.0	TKR/3435THR/2950	OA	All-cause mortality	*Normal weight (ref):***TKR**Underweight: 3.72 (1.94–7.08);Overweight: 0.79 (0.57–1.09);Class I obese: 0.66 (0.47–0.92);Class II obese: 0.54 (0.35–0.82);Class III obese: 0.73 (0.46–1.16)**THR**Underweight: 1.89 (0.76–4.71);Overweight: 0.84 (0.63–1.12);Class I obese: 0.86 (0.63–1.17);Class II obese: 0.77 (0.49–1.22);Class III obese: 0.68 (0.35–1.33)	Age, Australian-born, year of operation, gender, diabetes, primary diagnoses, and ASA category
Dowsey et al. 2018 [9]	USA	KPTJRR	67	4.0	TKR/109,333THR/57,049	OA	All-cause mortality	*Normal weight (ref):***TKR**Underweight:1.88 (1.33–2.64);Overweight: 0.76 (0.71–0.82);Class I obese: 0.71 (0.66–0.78);Class II obese: 0.73 (0.66–0.81);Class III obese: 0.91 (0.80–1.04)**THR**Underweight: 2.09 (1.65–2.64);Overweight: 0.73 (0.67–0.80);Class I obese: 0.68 (0.6–0.75);Class II obese: 0.71 (0.62–0.81);Class III obese: 0.91 (0.75–1.12)	Age, race, year of operation, gender, diabetes, bilateral, primary diagnoses, and ASA category
Evans et al. 2021 [11]	UK	NJR	NR	3.8	TKR/493,710	OA	90-day mortality	*Normal weight (ref):*Underweight: 1.64 (0.87–3.09); Overweight: 0.76 (0.65–0.90);Class I obese: 0.69 (0.58–0.82);Class II obese: 0.88 (0.72–1.09);Class III obese: 1.17 (0.90–1.54)	Age, sex, ASA grade, indication for operation, year of primary TKR, and fixation type

*ASA*, American Society of Anaesthesiologists; *CI*, confidence interval; *KPTJRR*, Kaiser Permanente Total Joint Replacement Registry; *NJR*, National Joint Registry for England and Wales; *NR*, not reported; *OA*, osteoarthritis; St Vincent’s Melbourne Arthroplasty, SMART; *THR*, total hip replacement; *TKR*, total knee replacement

The findings of higher mortality in the underweight individuals are not unexpected as this is seen in many other disease processes, which could be due to comorbidities and less “reserve” to fight illness. The reasons for the obesity paradox in many disease states, including vascular disease, remain uncertain. Some mechanisms have been proposed to explain the obesity paradox in HF and CVD and one of them is the role of cardiorespiratory fitness (CRF) (Lavie et al. 2016b; [40]. Cardiorespiratory fitness, an index of habitual physical activity, integrates human body function under demanding physiological states, reflects an individual’s functional capacity, and it is a major determinant of clinical outcomes [25]. A higher CRF is associated with improved outcomes in HF and other vascular and non-vascular diseases [7] as well as the reduced risk of these diseases [19, 22]. It has been suggested that having good CRF may offset the harmful consequences of excess body weight and other CVD risk factors, thereby enabling individuals to be “fat but fit” [26]. Furthermore, individuals with obesity typically have an increased amount of lean mass, which is associated with improved CRF [7]. Another reason is that because HF is associated with frailty, having more body mass and weight could be itself protective [30]. It is known that the frailty phenotype is predictive of increased morbidity, hospitalisation, and mortality [1]. Hence, the presence of additional adipose tissue in obese patients may serve as metabolic reserves for stressful events or critical illness. Unmeasured confounding induced by selection bias (termed collider bias) has been reported to be a partial explanation of the obesity paradox, but that it is unlikely to be the main explanation [45]. It has also been suggested that the obesity paradox in CVD could be a result of biases involving reverse causation and confounding by smoking [47]. Though the obesity paradox observed in patients undergoing joint replacement for OA could be due to confounding factors such as comorbidities and smoking, the consistency of the evidence cannot be ignored. The majority of reports did not provide data on the prevalence of CVD and other comorbidities or account for these in their analyses. Thornqvist and colleagues, in their analysis of Danish nationwide registries, provided detailed data on comorbidities including cardiovascular conditions and adjusted for these in their multivariable models, which did not diminish their associations [49]. Another potential explanation is that it appears the obesity paradox holds true when older age and some specific chronic diseases co-exist. The excess mortality associated with high BMI is known to decline with advancing age [5]; furthermore, the data consistently shows that underweight status confers an excess risk of death than overweight or obesity in old age, which could mask any deleterious effects of overweight or obesity. As in many conditions including CVD, the exact mechanism underlying the obesity paradox in joint replacement for OA is uncertain. It is not known if pre-existing CVD or other specific comorbidities mediate the obesity-related mortality among patients undergoing joint replacement for OA. Given that some studies of joint replacement in patients with OA have not reported the unusual relationship between BMI and mortality [42], it is crucial that future work investigates whether the obesity paradox really applies in this population. Studies need to provide detailed data on baseline comorbidities including CVDs in their reports and adequately account for these comorbidities and other potential confounders in their analyses in addition to sensitivity analysis using exclusions. We acknowledge the challenges involved in obtaining access to these data especially with regards to registry studies, due to complexities involved in data linkages, the high costs involved, and problems with missing data. Currently, there is no data to suggest if the obesity paradox extends to patients with early- to mid-stage OA or may have a wider applicability. However, there is emerging evidence on the existence of an obesity paradox in hip fracture patients undergoing surgery [36, 48]. Modig and colleagues [36], in their analysis of the association between BMI and survival after hip fracture, showed that overweight and obesity were associated with better survival compared with normal weight. The associations were not attenuated following adjustment for comorbidities and dementia; however, they demonstrated a more pronounced effect of the obesity paradox among patients who were oldest, confirming the suggestion that the obesity paradox may be linked with older age. There may be implications of clinical interest if the obesity paradox represents a true causal phenomenon in joint replacement for OA and other musculoskeletal conditions. Higher levels of fitness (as measured by CRF) are accompanied by a lower risk of CVD outcomes and prolonged life expectancy, across all levels of adiposity [3]. Several reports have also observed that the association of obesity with adverse outcomes is reduced on accounting for fitness levels [20]. If the increased mortality risk attributed to obesity can be attenuated with increased fitness, this could be translated into clinical benefits. This suggests that improving fitness levels (CRF) should be a priority for patients about to have joint replacement, even without apparent changes in BMI. A physically active lifestyle activity (particularly aerobic training) promotes good CRF levels. The evidence on the role of physical activity and good CRF in improving health outcomes and reducing chronic disease and mortality risk is overwhelming [22, 27, 28, 52]. There is even evidence to suggest that physical activity and good fitness levels reduce the risk, duration or severity of infectious diseases including COVID-19 (Brawner et al. 2021; [24]. Even standing, which is the least active behaviour, has been reported to be associated with health benefits compared with sitting [51]. However, the majority of populations do not achieve physical activity levels recommended by established guidelines — 150–300 min/week of moderate-intensity or 75–150 min/week of vigorous-intensity aerobic physical activity/exercise for adults [41]. In the general population, various strategies for promoting physical activity have been recommended and these include (i) individual level interventions; (ii) community-wide and school-based physical activity programmes; (iii) policy and environmental changes that improve access to physical activity; and (iv) information and communication technology, but levels are still low. For patients about to undergo hip or knee replacement, physical activities or exercises that are attractive and feasible for them, need to be identified and tailored to improve their fitness levels before surgery. However, support for the obesity paradox does not necessarily promote obesity; intentional weight loss has numerous health benefits and is advantageous in improving outcomes in joint replacement. Last but not the least, given the excess mortality risk associated with underweight status in patients undergoing joint replacement, it appears that more attention need to be given to this population group during preparation for surgery compared with overweight or obese patients.

## Data Availability

Not applicable.
